# Distinct Soil Microbial Communities in habitats of differing soil water balance on the Tibetan Plateau

**DOI:** 10.1038/srep46407

**Published:** 2017-04-12

**Authors:** Yuntao Li, Jonathan Adams, Yu Shi, Hao Wang, Jin-Sheng He, Haiyan Chu

**Affiliations:** 1 State Key Laboratory of Soil and Sustainable Agriculture, Institute of Soil Science, Chinese Academy of Sciences, East Beijing Road 71, Nanjing 210008, China; 2 University of Chinese Academy of Sciences, Beijing 100049, China; 3 Department of Biological Sciences, Seoul National University, Seoul, Republic of Korea; 4 Department of Ecology, College of Urban and Environmental Sciences, and Key Laboratory for Earth Surface Processes of the Ministry of Education, Peking University, Beijing 100871, China; 5 Key Laboratory of Adaptation and Evolution of Plateau Biota, Northwest Institute of Plateau Biology, Chinese Academy of Sciences, Xining 810008, China

## Abstract

Global change may be a severe threat to natural and agricultural systems, partly through its effects in altering soil biota and processes, due to changes in water balance. We studied the potential influence of changing soil water balance on soil biota by comparing existing sites along a natural water balance gradient in the Qinghai-Tibetan Plateau. In this study, the community structure of bacteria, archaea and eukaryotes differed between the different soil water conditions. Soil moisture was the strongest predictor of bacterial and eukaryotic community structure, whereas C/N ratio was the key factor predicting variation in the archaeal community. Bacterial and eukaryotic diversity was quite stable among different soil water availability, but archaeal diversity was dramatically different between the habitats. The auxotype of methanogens also varied significantly among different habitats. The co-varying soil properties among habitats shaped the community structure of soil microbes, with archaea being particularly sensitive in terms of community composition, diversity and functional groups. Bacterial and archaeal phylogenetic community turnover was mainly driven by deterministic processes while stochastic processes had stronger effects on eukaryotic phylogenetic community turnover. Our work provides insight into microbial community, functional group and phylogenetic turnover under different soil conditions in low-latitude alpine ecosystem.

Soil microorganisms play an essential role in global C and N cycles^1^,^2^,^3^ and experimental studies demonstrate that they have the potential to respond to environmental changes^4^,^5^,^6^. However, the capacity to set up experimental manipulation studies is limited, so in order to predict more generally how soil microbial systems will alter in response to environmental changes including those associated with global warming, it will be necessary to compare different points along existing environmental gradients as a proxy for future change over time^7^.

Previous studies have often found that pH is a major driving force for spatial and temporal variation in soil bacterial communities^8^,^9^,^10^,^11^,^12^. However, other factors, particularly moisture, also have strong effects on microbial communities^13^,^14^. This is very reasonable as soil water content helps to determine soil texture, bulk density, oxygen availability and connectivity within soils^2^,^15^,^16^, which can have major influence on microbial communities when pH hardly varied. The change in soil moisture condition mainly results from natural gradients or global climate change. Topographic study showed Mean annual precipitation was strongly correlated to the soil microbial basal respiration in semi-arid area^17^. Different soil moisture conditions have been found to cause changes in bacterial and fungal community structure, as well as the overall enzyme activity, in grassland, forest, alpine tundra and alkaline ecosystem^15^,^18^,^19^,^20^. Another way in which soil moisture effects may be of importance is the influence of climate change. There have already been various studies on how artificial warming can affects soil microbial community structure and processes^4^,^21^,^22^. The shifts in microbial community and physiology, in turn, may lead to changes of greenhouse gas fluxes emitted, either intensifying or mitigating the velocity of climate change^22^,^23^. One of the potential ways in which global warming might affect soil microbial communities and processes is through its effects on soil water balance, for example through melting of permafrost layers.

The effects of permafrost melting on microbial processes have already been investigated to some extent. As a major component of terrestrial ecosystems, permafrost is characterized by low temperature and low nutrient availability, but includes a major part of the global carbon pool^24^. Increase in atmospheric temperature will accelerate the thawing of permafrost, which can lead to wide-ranging consequences including alteration in soil properties including soil nutrient availability and plant community composition^16^,^25^,^26^. Located in the Northwest of China, the Qinghai-Tibetan Plateau is the largest permafrost area on the Eurasian continent with fairly high altitude and low latitude. It is estimated that this region has a soil organic carbon pool of 30–40 × 10^9^ t, accounting for 2–3% of the global soil organic carbon storage^27^. The plateau is relatively sensitive to climate change due to the higher mean annual temperatures and thinner permafrost layer compared with other high-latitude permafrost areas^28^. Recent warming has already been observed to have resulted in permafrost loss in the Qinghai-Tibetan Plateau. Additionally, the Qinghai-Tibetan plateau is calculated to have lost a total of 1.79 T g of its soil carbon storage during 1986 to 2000 in the 0–30 cm soil layer due to the degradation of the aboveground vegetation^29^. Permafrost thawing is likely to add to this carbon loss.

Besides the microbial community and diversity investigation, there are more attention paid to the ecological process in governing microbial phylogenetic community turnover. Beta NTI has been applied to find the relative role of deterministic and stochastic processes^30^. Plenty of study has showed that deterministic processes are the main ecological force that drive the turnover of bacteria in various environment^30^,^31^. However, there is little research analyzing the phylogenetic community turnover especially on Tibetan Plateau and only focusing on bacteria^32^. We know very little about the phylogenetic turnover pattern for archaea and eukaryotes on Tibetan Plateau. We conducted our study at Haibei Station in the Qinghai-Tibetan Plateau, to investigate the soil microbial communities not only for their diversity and community structure, but also we looked into the relative role of deterministic and stochastic processes that governing their phylogenetic turnover. The comparative study included three different habitats with different soil water conditions and three different groups of microbes (bacteria, archaea and eukaryotes). Given above, this experiment was aimed to answer: (a). What is the microbial structure of the three habitats and what are the driven environmental factors, (b). Do different kind of microbes have the same extent of variation among habitats in their diversity and community structure and (c). Do different kind of microbes share a same pattern in their phylogenetic community turnover?

## Results

### Soil biochemical properties across different vegetation

Soil chemical properties differed significantly among the three habitats (Table 1). Wetland had the highest soil moisture, C/N ratio, dissolved organic carbon and dissolved organic nitrogen while meadow had the lowest concentration. Marsh had the highest total carbon and total nitrogen and meadow remained the lowest. The pH showed little variation across the different habitats (7.6–8.3), although statistically significant differences were found.

### Microbial community composition

**Figure 1:**
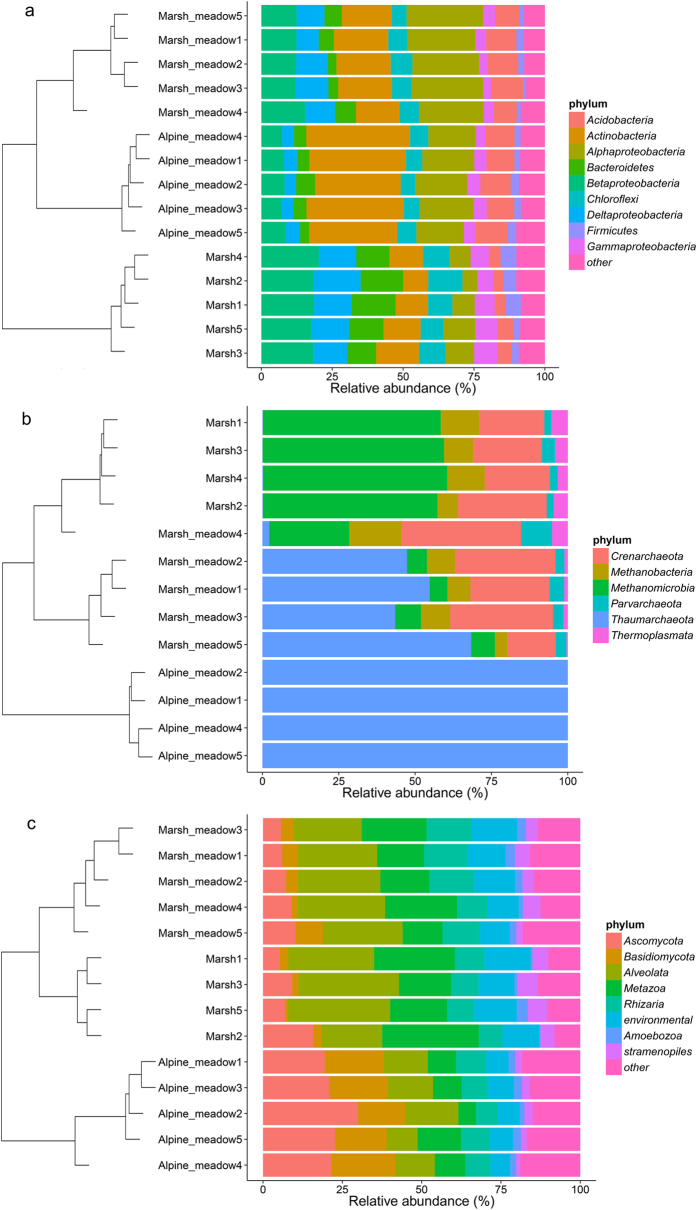
Relative abundances of the dominant microbial phyla in all samples at Haibei Station (**a**) bacteria; (**b**) archaea; (**c**) eukaryotes). Relative abundances are based on the proportion of sequences that could be classified at the phylum level. Samples were clustered using UPGMA method based on OTU table using bray-curtis dissimilarity.

Across all soil samples, we obtained 53,132 quality sequences of bacteria with 1,422–5,328 sequences per sample (mean 3,542), 87.7% classified, 172,014 quality sequences of archeae with 2,995–20,053 sequences per sample (mean 11,468), 97.9% classified and 68,948 quality sequences of eukaryotes with 1066–9,302 sequences per sample (mean 4,925), 88.7% classified. *Actinobacteria, Alphaproteobacteria, Acidobacteria, Betaproteobacteria, Chloroflexi, Bacteroidetes* and *Deltaproteobacteria,* accounting for more than 80% bacterial sequences, were the dominant phyla of bacteria while *Gammaproteobacteria and Firmicutes* were also relatively abundant. More than 95% of total archaeal sequences were classified to *Thaumarchaeota, Euryarchaeota* and *Crenarchaeota*, with the remaining sequences classified to a newly found phylum *Parvarchaeota*. The total eukarya included *Fungi, Alveolata, Metazoa* and *Razaria* as its most abundant lineages, which accounting for more than 70% of the eukaryotic sequences followed by the less abundant phyla *moebozoa* and *Stramenopiles* (Fig. 1a–c). In total, 29 bacterial, 4 archaeal and 12 eukaryotic phyla were detected (Tables S2–S4).

**Figure 2:**
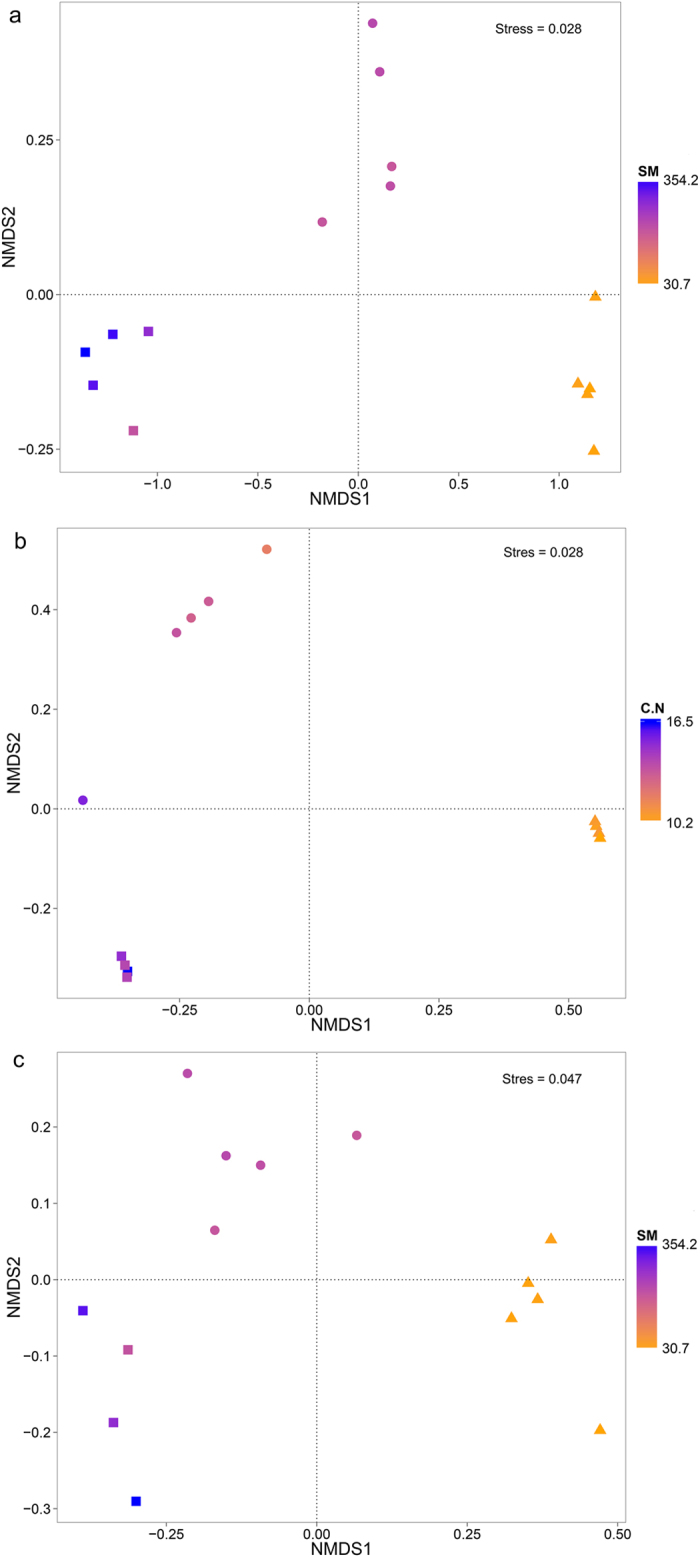
Microbial community (**a**) bacteria; (**b**) archaea; (**c**) eukaryotes) compositional structure in different habitats at Haibei Station calculated by non-metric multi-dimensional scaling (NMDS) using Bray-Curtis dissimilarity (▲ Alpine meadow; ● Marsh meadow; ■ Marsh). Environmental factors that had the most explanation to microbial community variation in DistLM analysis were fitted to the plots and color gradient indicate the value of the factor.

The microbial communities differed dramatically across vegetation type (Fig. 2). Pair-wise comparison showed that there were significant differences between each pair of vegetation types among all three kinds of microbes (Table S5). Whole community variation among different vegetation types showed that archaea community had much larger variation than bacteria and eukaryoes where bacteria community variation was slightly higher than eukaryotic community (Table S6). Relative abundance of dominant phyla showed significant differences between bacteria, archaea and eukaryotes (Fig. S2a–S2c). Amongst the bacteria, the relative abundance of *Betaproteobacteria, Chloroflexi, Bacteroidetes, Deltaproteobacteria, Gammaproteobacteria* and *Firmicutes* were dramatically higher in wetland, two of which (*Betaproteobacteria* and *Bacteroidetes*) were also significantly higher in Marsh than Meadow. However, there was significantly lower relative abundance of *Actinobacteria, Alphaproteobacteria* and *Acidobacteria* in marsh and wetland, compared with Meadow (Fig. S2a). Amongst archaea, meadow had the highest relative abundance of *Thaumarchaeota* whereas marsh had the highest relative abundance of *Euryarchaeota* and notably, there was no single dominant phylum in the marsh meadow habitat, in contrast to the absolute dominance of *Thaumarchaeota* in meadow and *Euryarchaeota* in marsh (Fig. S2b). Amongst the Eukarya, the relative abundance of *Alveolata, Metazoa, Razaria* and *Stramenopiles* significantly increased along the moisture gradient while *Amoebozoa* abundance showed no significant differences. Interestingly, from being the dominant group in meadow, the fungal relative abundance was much lower Marsh and Wetland (Fig. S2c).

Microbial communities co-varied with several soil chemical variables. Mantel tests showed significant correlations between pH, soil moisture, TC, TN, C/N ratio, DOC, DON and NH_4_^+^ and microbes community (Table S7). Of all the environmental variables examined, soil moisture showed the highest correlation with the community composition both in bacteria (r = 0.792, P = 0.001) and eukaryotes (r = 0.834, P = 0.001). In archaea, soil moisture, total carbon, total nitrogen and C/N ratio all showed high yet similar correlation, with total carbon to be the highest (r = 0.684, P = 0.001). Thus, DistLM were applied to test the variation of microbial community explained by every environmental factor (Table S8). The results of DistLM indicated that soil moisture explained the most variance among all factors in bacterial and eukaryotic community, which was in correspondence with the result of Mantel tests. Furthermore, C/N ratio was found to explain the most variance in archaeal community. Notably, soil moisture had no explanation on the variation of archaeal community revealed by DistLM. It may be because the colinearity of soil moisture and other factors. The factors that significantly correlated with microbial community composition were tested by VIF and factors with VIF value less than 20 were selected to perform CCA (Fig. S3a–S3c). CCA demonstrated that soil moisture had the strongest impact on bacterial and eukaryotic community composition and C/N ratio had the strongest effect on archaeal community. Meanwhile, pH, TC, TN and DOC also had significant but less impacts on microbial community composition. Thus, bacterial and eukaryotic community composition were best predicted by soil moisture, while C/N ratio was the fundamental factor that could predict variation in archaeal community composition amongst the various habitat types at Haibei Station.

### Microbial diversity

**Figure 3:**
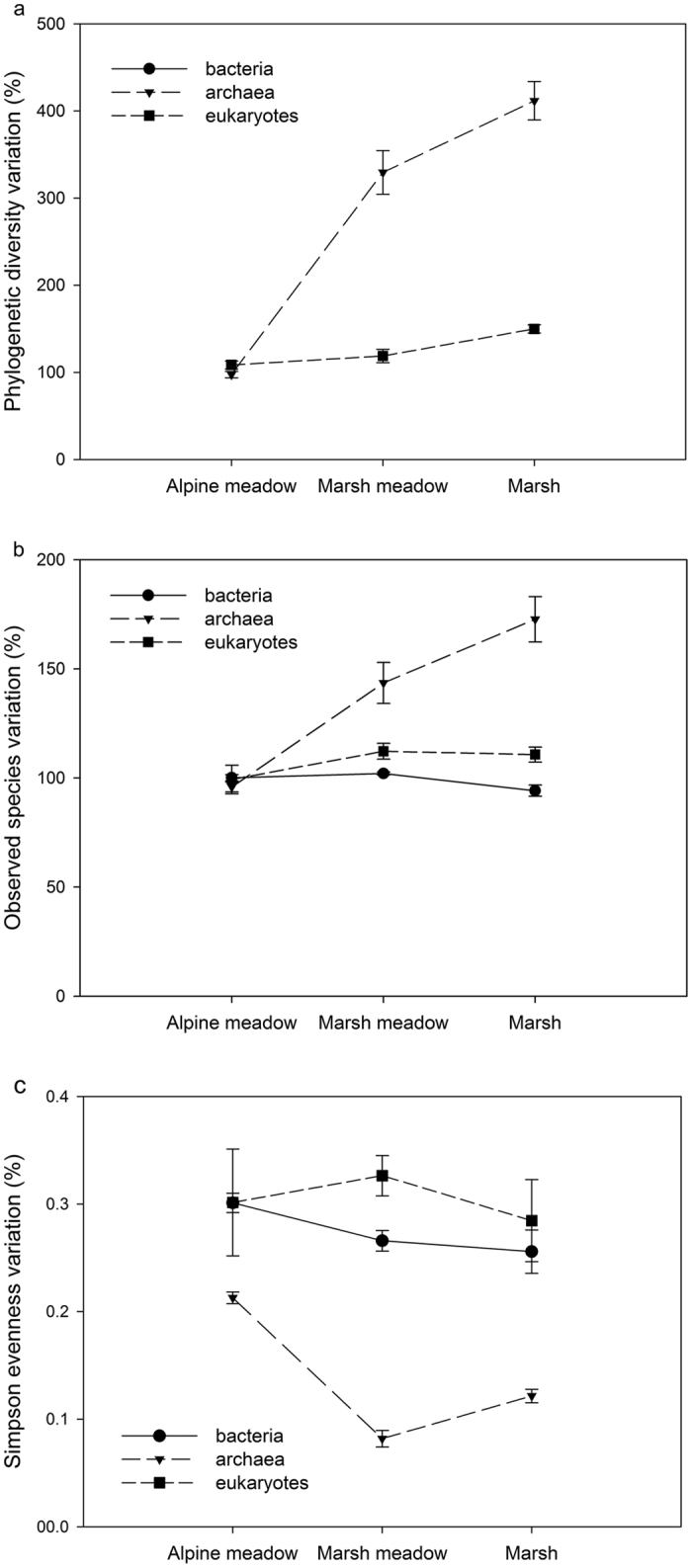
Microbial alpha diversity variation (**a**) phylogenetic diversity; (**b**) observed species; (**c**) Simpson evenness) across vegetation types. PD and OTUs variation were determined by dividing diversity value of each sample by that of the first sample in Alpine meadow. All Diversity indices were calculated using a random subset of 2800, 1110 and 640 sequences per sample, respectively. Error bar stands for standard error.

Phylogenetic diversity, observed species richness and Shannon Index were investigated for all sites (Table S9). For bacteria, both observed species richness and Shannon Index were significantly lower in the wetland habitat. For phylogenetic diversity, meadow was instead the least diverse. For archaea, all of three tested diversity indices showed a similar pattern, with marsh and/or wetland having significantly higher diversity than meadow. As for Eukarya, only wetland had higher microbial diversity. It is noteworthy that there was much more fluctuation in archaeal diversity between habitat types than was the case for bacteria and Eukarya (Fig. 3). This may indicate that archaea are particularly sensitive to changes in moisture conditions, as might be expected due to changes in abundance and diversity of methanogenic archaea.

We correlated microbial diversity with soil characteristics (Table S10). For bacteria, phylogenetic diversity was positively correlated with total carbon and total nitrogen. Amongst archaea, diversity was positively correlated with soil moisture, total carbon, total nitrogen, C/N ratio, dissolved organic carbon and dissolved organic nitrogen. For eukaryotes, diversity positively correlated with soil moisture, C/N ratio and dissolved organic carbon. Archaeal species richness was positively correlated with soil moisture, C/N ratio and dissolved organic carbon whereas eukaryotic species richness was positively correlated with total carbon and dissolved organic carbon. No significant correlation was found in eukaryotes.

**Figure 4:**
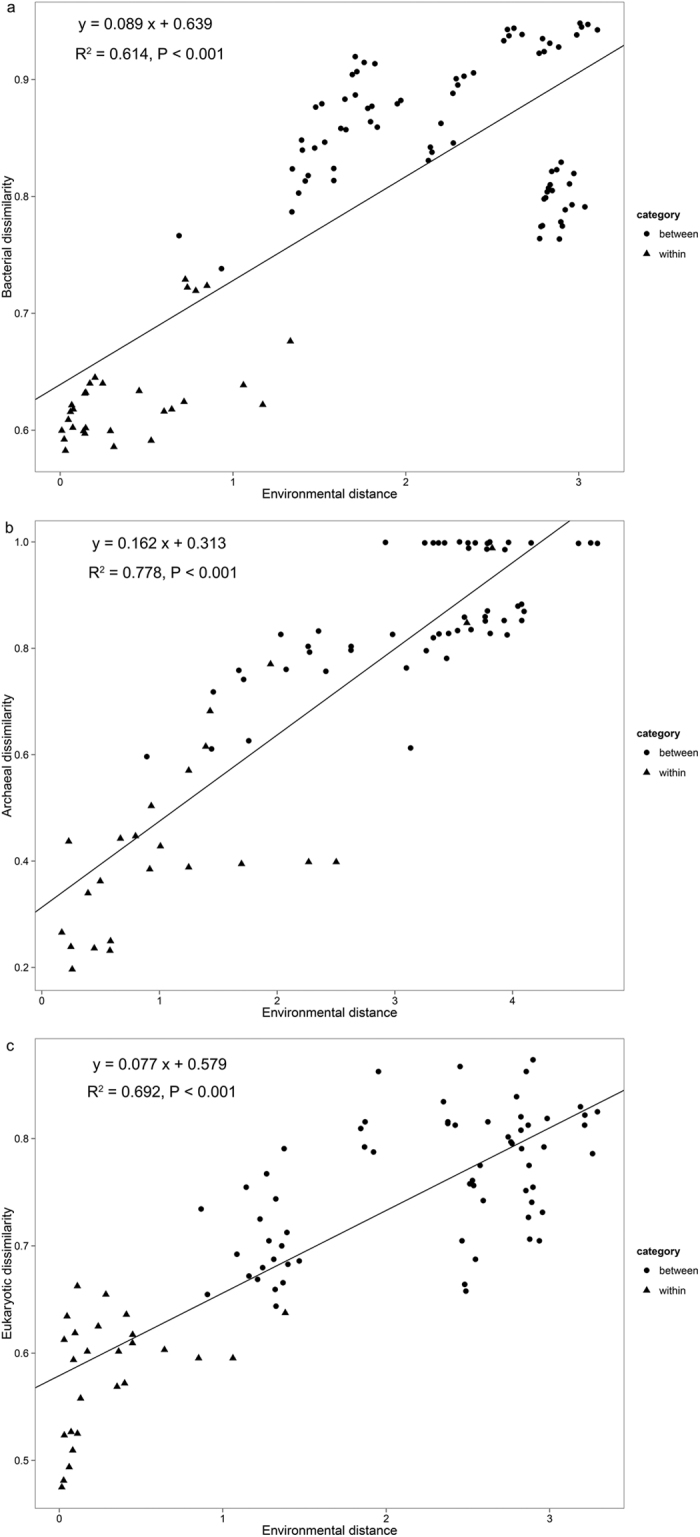
Regression of environmental distance and microbial dissimilarity (**a**) bacteria; (**b**) archaea; (**c**) eukaryotes). Environmental distance was calculated using euclidean dissimilarity and only factors that were significantly correlated to microbial community variation were chosen to perform the calculation (that is, SM and TN for bacteria; C/N, TC, DOC and TN for archaea; SM and TC for eukaryotes). Microbial dissimilarity was calculated using bray-curtis dissimilarity. Between: plots pair belonging to different habitats; within: plots pair in same habitats.

Linear Regression was applied to find the relatedness between microbial beta diversity and environmental distance between plots (Fig. 4). Microbial beta diversity were determined by Bray-Curtis dissimilarity based on OTU table and environmental distance was calculated by Bray-Curtis dissimilarity using the normalization value of environmental viriables that were significantly correlated with community variation in Mantel test and DistLM. Results showed that microbial beta diversity was linearly well scaled with environmental distance (R^2^ ≥ 0.638). Bray-Curtis dissimilarity was categorized into “within-site pairs” and “between-sites pairs” and the result showed that microbial community was more similar within the same site and more divergent in different habitats (Fig. S4).

### Microbial community function

Different indicator functional pathways of bacteria community was found among three habitats (Table S11). Metabolism and cellular processes related pathways was found in all habitats as indicators, however marsh had a fairly low level pathway gene numbers. Organismal Systems related pathways was found in alpine meadow and marsh meadow as indicators. Environmental information processing and genetic information processing pathways was only found significantly higher in alpine meadow than the other two habitats. Besides the predictive bacterial function group, actual functional taxon also showed significant changes. The family Rhizobiales differed dramatically among habitats (Fig. S4)

Methanogenic archaea auxotype was determined (Fig. S6). The result showed that no methanogenic archaea was found in alpine meadow whereas several kinds of auxotype were observed in both marsh meadow and marsh. Notably, relative abundance of hydrogenotrophic, facultative and obligate acetoclastic methanogens was significantly higher in marsh than in marsh meadow, as well as the ratio of acetoclastic and hydrogenotrophic methanogens.

### Processes governing microbial community turnover

**Figure 5:**
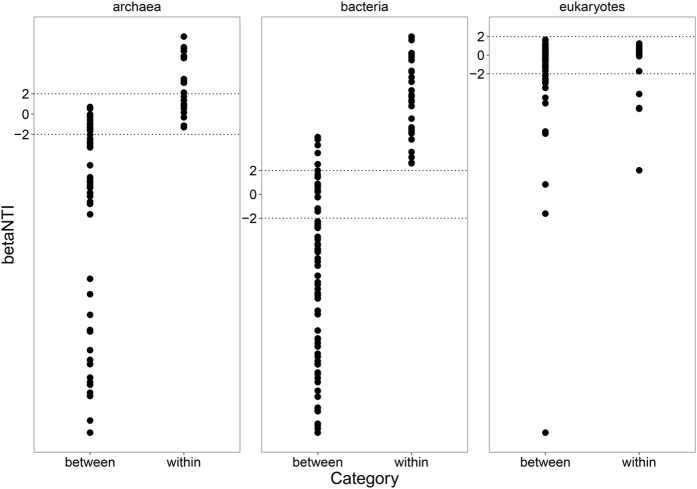
Microbial phylogenetic community turnover pattern tested by beta NTI. −2 and +2 were the ecological processes threshold for deterministic and stochastic force. Between: comparison of different habitats; withi: comparison in same habitats.

For bacteria, 15.24% of the beta NTI value was between −2 and +2, 33.33% above +2 and 51.43% below −2, with an average of −2.30. For archaea, 51.28% of the beta NTI was below −2, 35.90% between −2 and +2 and 12.82% above +2 with an average of −6.06. As for eukaryotes, 79.12% of the beta NTI value was between −2 and 2 with an average of −1.51 (Fig. 5; Table S12). Within and between habitats beta NTI was calculated. In between habitats comparisons, the average beta NTI were −6.63, −9.44 and −1.83 for bacteria, archaea and eukaryotes, respectively. Detailed between habitats results showed that there were significant differences among different site pairs. The pair AM-MA (alpine meadow vs. marsh) has the lowest beta NTI value among all between pairs (Fig. S7; Table S13).

## Discussion

### Microbial community and co-varied environmental factors

Combining the landscape-scale gradient sampling strategy with high-throughput sequencing results, we demonstrated that microbial community (in terms of bacteria, archaea and eukaryotes) varied significantly between the three sets of soil water conditions on the Qinghai-Tibetan Plateau. It is indicated that in our study area, soil moisture was the main driving factor of bacterial and eukaryotic community, whereas the archaeal community was mainly affected by C/N ratio (Fig. 2; Tables S7 and S8; Fig. S3a–S3c). This pattern is similar to those found in previous studies^15^,^33^,^34^,^35^,^36^, and may be caused by the physiology of microbes and differing nutrient availability among habitats. Many bacteria and almost all eukaryotes are aerobic organisms, and lack of oxygen can strongly alter their community composition. For instance, the relative abundance of fungi was lower in the marsh meadow and wetland, since fungi are generally intolerant of low oxygen environments^28^. The increase of DOC and DON gave bacteria and eukaryotes more access to soil available nutrients, which will also change their community^37^,^38^. C/N ratio is commonly regarded as an indication of soil nutrient structure, thus we owe the strong correlation between C/N ratio and archaeal community to the nutrients variation instead of C/N ratio itself 34.

The driving effect of soil pH on microbial community has been found on various scales from continental to submeter scale^8^,^9^,^10^,^39^. Nevertheless, in our study the soil pH varied by only a small range between different habitats, although differences between the habitats were statistically significant. The relative abundance of *Acidobacteria* was significantly lower in wetland than meadow and marsh meadow, however the pH variation among habitats did not present the same pattern, indicating that pH may not play a major role in structuring the microbial community. Instead, our study indicated that the main driving factor in shaping the microbial community was soil moisture and C/N ratio, as well as TC, DOC and TN, which have a strong connection with soil nutrient conditions. The sampling site was a natural landscape and has not been influenced by mankind activity. Since there is a slight slope across the three ecosystems, when it rains, the rainfall will accumulate at the bottom of the slope and cause the soil water content to increase, which is the position of marsh. On Haibei Station, there are many similar slopes that have alpine meadow on the top of them, marsh meadow in the middle and marsh at the bottom. Thus, the change in soil nutrient conditions is due to the result of geography and physical progress^40^. Combining all above, although soil moisture and C/N ratio were found to be the main factors that shaped microbial community structures, other edaphic factors also had significant influence. Microbial community variation was affected by the multiple co-varied environmental factors among different habitats rather than one certain driver.

We also found dramatic differences in microbial functional community among different habitats (Table S11; Figs S5 and S6). In the bacterial functional community predicted by PICRUSt, the Environmental Information Processing and Genetic Information Processing pathways was only enriched in alpine meadow. This indicate that bacterial community has more interaction with its surrounding environment and was more active in transcription and translation processes. As shown above, alpine meadow consists of various plant species and some of them such as Leguminosae, build symbiosis with certain bacterial group^41^. So the connection between bacteria and environment may be due their interaction with upgound plants. With more nutrients allocated from above ground to underground, bacterial community gains more access to available carbon and nitrogen sources, which lead to faster growth and cell replication. As for archaeal functional groups, we also observed significant changes among the three vegetation types. There are hardly any methanogens observed in alpine meadow. For methanogens, there is trace amount of methanogens in alpine meadow (0.036% in alpine meadow compared with 18.3% in marsh meadow and 62.3% in marsh). Thus, there existed anaerobic microbes in alpine meadow, even though at a very low abundance. This is because the high soil heterogeneity that create anaerobic microenvironment for such microbes to grow. However, compared to other anaerobic microbes like Clostridia (0.18%, 0.88% and 2.9% in alpine meadow, marsh meadow and marsh, respectively), the variation of methanogenic archaea was larger. We assume this is not only related to oxygen condition, but also because of the characteristic of methanogenic archaea. This kind of microbes can only grow using limited substrate such as acetate and methyl compound. Natural acetate was produced by acetogens, which was a kind of bacteria that produce acetate only in low temperature. As alpine meadow lacks the ability to maintain soil temperature at a relatively low level, thus there is hardly any acetogens in alpine meadow and this would result in the lack of substrate for methanogenic archaea to grow. In our result, the relative abundance of Genus Acetobacterium was 0%, 0.025% and 0.64% in alpine meadow, marsh meadow and marsh, respectively. So we assume the oxygen and lack of substrate together affect the distribution of methanogens. It has also been investigated that two thirds of methane produced through methanogens comes from acetoclastic methanogens^42^. Thus, we predict methanogenesis in marsh would be much stronger than in marsh meadow since there was a large proportion of acetoclastic methanogens in marsh. The reason why acetoclastic methanogens are more abundant in marsh than in marsh meadow still needs further study.

### Microbial alpha diversity and community stability

Reflecting ecosystem stability and functionality, microbial alpha diversity reveals valuable information about the state of the microbial community. We investigated both richness and evenness to assess the possible stability and functional redundancy of microbes. Our results showed that diversity of bacteria and eukaryotes changed only a little among habitats (Fig. 3; Table S9). Previous research also reached parallel conclusions^43^. Although the overall diversity did not varied much, groups that are closely associated with plants showed significant changes. For example, relative abundance of Rhizobiales hardly changed in alpine meadow and marsh meadow but decreased sharply in marsh (Fig. S5). This correspond to the vegetation diversity pattern of these habitats that marsh barely has upgound plants species. Thus, we believe that vegetation cover played a major role in determining the diversity of bacteria and eukaryotes, especially those considered to be closely associated with plant. However, archaeal diversity was correlated with various edaphic properties, as well as a sharp rise in richness and change in evenness along the water gradient (Fig. 3; Table S10). In term of community structure, archaea also had larger variation than bacteria and eukaryotes. These results indicated that archaeal diversity was sensitive to soil moisture condition and its consequential influence on soil properties. We believe this pattern ascribed to soil oxygen availability in that methanogens favor anaerobic environment whereas Thaumarchaeota need oxygen to live. There is little evidence of archaea forming any strong associations with other organisms, despite speculative statements^44^,^45^. Thus external environmental factors seem likely to be more important than biotic factors.

These results implied that different microorganisms may have distinct distribution patterns, and that it is more appropriate to draw conclusions by investigating multiple groups of microbes. In this study, we found marsh meadow forms a transition zone between alpine meadow and marsh. Its soil properties and archaeal functional community characteristics was more similar to marsh, however, its vegetation composition and bacterial functional groups was more close to the alpine meadow.

### Microbial beta diversity and ecological processes

Through the linear regression, we illustrate that plots that are more dissimilar to in terms of environmental distance (such as between sites) also harbor microbial communities that are more dissimilar to each other (Fig. 4). Plant beta diversity has been found to predict microbial beta diversity at a continental scale^46^. Here we showed other than plant cover, environmental distance can also predict microbial beta diversity at a landscape scale. We determined the environmental distance by taking all factors that are significantly correlated with community variation into consideration instead of one factor that has the highest correlation coefficient. Thus, this environmental distance model is much more persuasive in predicting microbial beta diversity. Notably, within-site pairs showed lower beta diversity compared with between-site pairs (Fig. S4). This is quite reasonable as the microbes have a strong ability to disperse widely^47^ and the microbial community would be similar given a relatively consistent environmental stress.

Within a single habitat in the landscape-scale region, neither environment nor geographic distance could have significant influence on microbial distribution. Furthermore, short generation times may allow microbes to settle down in a short period instead of being eliminated by native organisms. However, when it comes to the site level, even when microbes are capable of traveling from site to site, environment begin to intervene. Environmental factors limit microbes to living in certain niches and shaped distinct microbial community in different ecosystems. Our landscape-scale results are capable of explaining the behavior of microbial random dispersion and the impact of environmental stress on microbes simultaneously.

As a whole, our findings provide detailed information about microbial phylogenetic community turnover pattern at landscape-scale on Tibetan Plateau, especially concerning different soil water availability. Our study observed that bacterial and archaeal phylogenetic community turnover were more influenced by deterministic process while stochastic process has dominant impact on eukaryotic phylogenetic community turnover. There has been several study focusing on bacterial phylogenetic turnover, which revealed that deterministic processes has the major impact^30^,^31^,^32^ and our finding was in consistent with the former research in bacteria. However, there is little study on archaeal and eukaryotic phylogenetic turnover, especially on Qinghai Tibetan Plateau. Our results indicated that archaeal phylogenetic turnover pattern was similar to that of bacteria but eukaryotic phylogenetic turnover was a quite different pattern, which was strongly governed by stochastic processes. For turnover between different habitats, our result showed that, for all kind of microbes, the beta NTI in pair AM-MA was significantly lower than in other pairs, indicating the strongest environmental filtering effect in microbial turnover from alpine meadow to marsh. This may be due to the natural characteristics of the three habitats. The water balance on alpine meadow and marsh was relatively stable so that we can observe a clear deterministic turnover pattern through these two habitats. But for marsh meadow, it is a semi-flooded habitat, which suffered from flooding and drying through the year. Thus, the marsh meadow was a typical habitat with frequent disturbance. According to the framework of neutral and deterministic processes^48^, when community suffered from disturbance, relative influence of stochasticity may increase due to the relaxation of selection. Here in our study, there was no beta NTI distributing between −2 and +2 for bacteria and archaea in pair AM-MA but some points in −2 to +2 in AM-MM and MM-MA, which means due to the disturbance in marsh meadow, stochastic processes has a second but important influence. We even observed major effects of stochasticity in archaea for MM-MA. This is also true for eukaryotes when tested by ANOVA even though stochastic processes govern its community turnover. The average beta NTI value for AM-MA was −5.21 and significantly lower than other between pairs, which showed strong deterministic processes in its turnover form alpine meadow to marsh.

## Methods

### Site selection and soil sampling

Our study site, the Haibei Station, was located on the northeast of Qinghai Province, China (37°29′-37°45′N, 101°12′-101°23′E). The area has a plateau continental climate, with elevation ranging from 2900–4000 meters, average annual temperature of −1.7 °C and average annual precipitation from 426–860 mm (Fig. S1a). Haibei Station is dominated by meadow vegetation (see below for description), but also contains alpine meadow, marsh meadow and marsh (Fig. S1b,S1d). We choose a continuum in geography containing the three vegetation types as our sampling site at an elevation of 3200 m. They formed a slight slope, on the higher side was the alpine meadow and on the lower side was the marsh. However, there is hardly elevation variations since the slope is very small. The three vegetation types mainly differ in their soil water availability and because of the slope, water will naturally accumulate the most at marsh site and the least at alpine meadow. The soil water balance was determined by its soil moisture, which was 32%, 207% and 301% for alpine meadow, marsh meadow and marsh, respectively (Table 1). We investigated the upground vegetation community composition of three habitats and got the species presence-absence data (Table S1). Meadow habitat at Haibei Station was dominated by Compositae (Ajania tenuifolia, Taraxacum mongolicum, Aster tataricus, Saussurea superba), Gramineae (Elymus nutans, Ptilagrostis concinna), Cyperaceae (Lancea tibetica, Kobresia humilis), Leguminosae (Medicago ruthenica, Tibetia himalaica), Ranunculaceae (Anemone cathayensis, Thalictrum rutifolium), Rosaceae (Potentilla anserina, Potentilla bifurca) and Bryophytes. Marsh meadow mainly consisted of Compositae (Saussurea romuleifolia, Aster tataricus) and Cyperaceae (Scirpus distigmaticus, Carex spp.). Vegetation composition in the marsh areas was fairly simple where only two species were found dominant (Carex spp., Heleocharis dulcis). To conduct our investigation into the impact of changing moisture conditions on soil microbes, we compared the steady state conditions of meadow, marsh and wetland. Study sites were located on a single mountain slope areas which contained all of these habitats.

#### Soil samples were collected on September 12th, 2012 in Haibei Station

Each habitat was divided into 5 plots as replicates. In each plot, soil was collected from nine points at a depth of 0–10 m and then mixed as one sample. In total, 15 samples were collected. After sampling, the soils were kept in a cooler and shipped refrigerated to the lab. The samples were thoroughly mixed and sieved to remove grassroots and stone, and divided into two parts: one part was stored at 4 °C for biogeochemical analysis; the other was stored at −40 °C for DNA analysis.

### Soil nutrients and microbial biomass analyses

Soil moisture was measured by repeatedly drying 5 g fresh soil until the soil reached a constant weight and then measuring the weight ratio of dried soil and evaporated water. Soil pH was measured after shaking a soil water (1: 5wt/vol) suspension for 30 min. Total carbon (TC) and total nitrogen (TN) were determined by dichromate oxidation and titration with ferrous ammonium sulfate. Dissolved organic carbon (DOC) and dissolved organic nitrogen (DON) were determined by flow analyzer.

### Soil DNA extraction

0.5 g sieved soil was used to extract soil total DNA using FastDNA^®^ SPIN Kit for soil (MP Biomedicals, Santa Ana, CA) according to the manufacturer’s instructions. The extracted soil DNA was dissolved in 60 μl TE buffer, quantified by NanoDrop and stored at −20 °C.

Microbial rRNA gene amplification. An aliquot (50 ng) of soil DNA was used as template for amplification. As for bacteria, the V4–V5 hyper-variable regions of the bacterial 16 S rRNA genes were amplified with primer set: 515 F: GTGCCAGCMGCCGCGG with a unique 7-bp barcode sequence; 907 R: CCGTCAATTCMTTTRAGTTT. Each sample was amplified in triplicate in a 50 μl reaction system under following condition: initial denaturation at 94 °C for 5 min; 35 cycles of denaturation at 94 °C for 45 s, annealing at 55 °C for 45 s, and extension at 72 °C for 45 s; final extension at 72 °C for 10 min. As for archaea, the V3–V5 hyper-variable regions of the archeael 16 S rRNA genes were amplified with primer set: ARC344F: ACGGGGYGCAGCAGGCGCGA with a unique 7-bp barcode sequence; ARC915R: GTGCTCCCCCGCCAATTCCT^49^. Each sample was amplified in triplicate in a 50 μl reaction system under following condition: initial denatiration at 94 °C for 5 min; 35 cycles of denaturation at 94 °C for 30 s, annealing at 56 °C for 30 s, and extension at 72 °C for 1 min; final extension at 72 °C for 7 min. As for eukaryotes, the general eukaryote primer set Euk1A and Euk516R were chosen to amplify the eukarotic 18 S fragment. The primer set was: Euk1A: CTGGTTGATCCTGCCAG with a unique 7-bp barcode sequence; Euk516R: ACCAGACTTGCCCTCC. Each sample was amplified in triplicate in a 50 μl reaction system under following condition: initial denatiration at 94 °C for 5 min; 35 cycles of denaturation at 94 °C for 30 s, annealing at 56 °C for 30 s, and extension at 72 °C for 30 s; final extension at 72 °C for 7 min. Each primer set was modified by adding the Roche 454 ‘A’/‘B’ pyrosequencing adapter in order to be able to perform the pyrosequencing.

### 454 Pyrosequencing

PCR products were pooled together and purified by Agarose Gel DNA purification kit (TaKaRa). An equal amount of PCR product for each sample was combined in a single tube and run on Roche FLX 454 pyrosequencing machine (Roche Diagnostics Corporation, Branford, CT, USA), producing reads from the forward direction of F515, ARC344F and Euk1A for bacteria, archaea and eukaryotes, respectively.

### Processing sequencing data

Sequencing data were processed using Quantitative Insights Into Microbial Ecology 1.8.0 pipeline^50^. Sequences were filtered by quality and length control (Phred score threshold 25, length range from 200 bp to 600 bp) and then demultiplexed. After removing barcodes and primers, all sequences that passed quality control were denoised to be ready for sequence clustering. Next, sequences were clustered into operational taxonomic units (OTUs) at 97% simmilarity threshold using uclust method and representative sequence was chosen based on the most abundant sequence in each OTU. Taxonomy was assigned to each OTU through their representative sequence using the ribosomal database project (RDP) classifier against Greengenes database for bacteria and archaea and Silva database for eukaryotes^51^. Representative sequences were aligned using PyNAST and the aligned sequences were used to build the phylogenetic tree^52^. Rarefaction was applied to avoid the impact of different sequencing depth. A randomly selected subset of 2800, 1110 and 640 sequences per bacteria, archaea and eukaryotes sample was constructed to compare difference among samples. Rarefaction values were determined in order to retain the most sequences. Microbial alpha diversity index (Faith’s PD, Observed species, Chao1, Shannon and Simpson evenness) and beta diversity index (Bray-curtis dissimilarity) were calculated in QIIME.

### Statistical analysis

The significance of difference of taxonomic diversity among vegetation features were tested using SPSS 20.0. UPGMA clustering of samples was done in QIIME based on OTU table. R 3.1.0, with vegan package, was used to carry out multivariate statistical analysis^53^,^54^. Bray-Curtis dissimilarity based Non-metric multidimensional scaling (NMDS) was applied to visualize distance among samples. ADONIS (Multivariate ANOVA based on dissimilarities) was applied to test the significance of pairwise microbial community difference. Mantel tests were used to identify the correlation between environmental factors and community structure. All soil properties were tested by variance inflation factor (VIF) to judge the colinearity among the factors. Factors with VIF value less than 20 were selected to perform canonical correspondence analysis (CCA). DISTLM_forward3 were used to determine the significance and explained variance of each environmental factor^55^. Phylogenetic Investigation of Communities by Reconstruction of Unobserved States (PICRUSt) was used to predict the bacterial community functions online (http://huttenhower.sph.harvard.edu/galaxy). Metagenome was predicted through KEGG and functional pathway was summarized^56^. Indicator species analysis was applied to the functional pathways to find out the indicator pathways among different habitats. Auxotype of methanogenic archaea was determined based on their taxonomy annotation and previous study on these lineages^57^,^58^. Microbial phylogenetic community turnover was analyzed by beta NTI using Phylocom^59^. Pictures were illustrated in R and Sigmaplot 9.0 and modified in Photoshop CC. Maps were generated in R with package maptools^60^ and ggplot2^61^.

### Data availability

The nucleotide sequence data generated in this study are available in the SRA databases in The National Center for Biotechnology Information (NCBI) under the accession numbers SRP047360, SRP047361, SRP047400 for bacteria, archaea and eukaryotes, respectively.

## Additional Information

**How to cite this article:** Li, Y. *et al*. Distinct Soil Microbial Communities in habitats of differing soil water balance on the Tibetan Plateau. *Sci. Rep.* **7**, 46407; doi: 10.1038/srep46407 (2017).

**Publisher's note:** Springer Nature remains neutral with regard to jurisdictional claims in published maps and institutional affiliations.

## Supplementary Material

Supplementary Information

## Figures and Tables

**Table 1:** Biogeochemical properties among vegetation types at Haibei Station.

soil variable	Alpine meadow	Marsh meadow	Marsh
pH	8.3 (0.05)^a^	7.6 (0.41)^b^	7.7 (0.39)^ab^
SM (%)	32 (3)^c^	207 (12)^b^	301 (62)^a^
TC (%)	5.7 (0.3)^c^	23 (1.3)^a^	16 (1.7)^b^
TN (%)	0.54 (0.03)^c^	1.7 (0.18)^a^	1.1 (0.11)^b^
C/N	10.5 (0.2)^b^	13.4 (1.3)^a^	14.6 (1.1)^a^
DOC (mg/kg)	500 (274)^b^	859 (78)^ab^	1385 (467)^a^
DON (mg/kg)	9.8 (0.9)^b^	34 (7)^a^	36 (20)^a^
NO_3_^−^ (mg/kg)	13 (8.9)^a^	19 (20)^a^	3.3 (1.5)^a^
NH_4_^+^ (mg/kg)	6.5 (3.3)^b^	18 (9.3)^a^	16 (4.9)^a^

The values in brackets represent the standard deviation. Different letters represent significant differences. Abbreviations are SM: soil moisture; TC: total carbon; TN: total nitrogen; C/N: carbon/nitrogen ratio; DOC: dissolved organic carbon; DON: dissolved organic nitrogen.
