# Supplementary material for: Distinct Soil Microbial Communities in habitats of differing soil water balance on the Tibetan Plateau
Source: Sci Rep. 2017 Apr 12;7:46407. doi: 10.1038/srep46407 (PMC5388882; doi:10.1038/srep46407)

**Distinct Soil Microbial Communities at habitats of differing soil water balance on the Tibetan Plateau**

**Author names**: Yuntao Li^1, 5^, Jonathan Adams^2^, Yu Shi^1^, Hao Wang^3, 4^,Jin-Sheng He^3, 4^, Haiyan Chu^1,*^

**Affiliation**: ^1^ State Key Laboratory of Soil and Sustainable Agriculture, Institute of Soil Science, Chinese Academy of Sciences, East Beijing Road 71, Nanjing 210008, China.

^2^ Department of Biological Sciences, Seoul National University, Seoul, Republic of Korea.

^3^ Department of Ecology, College of Urban and Environmental Sciences, and Key Laboratory for Earth Surface Processes of the Ministry of Education, Peking University, Beijing 100871, China.

^4^ Key Laboratory of Adaptation and Evolution of Plateau Biota, Northwest Institute of Plateau Biology, Chinese Academy of Sciences, Xining 810008, China.

^5^ University of Chinese Academy of Sciences, Beijing 100049, China.

**^#^Corresponding author**: Haiyan Chu

**Corresponding address**: State Key Laboratory of Soil and Sustainable Agriculture, Institute of Soil Science, Chinese Academy of Sciences, East Beijing Road 71, Nanjing 210008, China

Tel.: +862586881356, Fax: +8602586881000, E-mail: hychu@issas.ac.cn

Table S1 Plant observed species among vegetation type. Plant species names were showed in binomial nomenclature. Numbers under vegetation names stands for replicates. “P” represents presence of the species, where “A” represents absence.

| Plant species | Alpine meadow | | | | |  | Marsh meadow | | | | |  | Marsh | | | | |
| --- | --- | --- | --- | --- | --- | --- | --- | --- | --- | --- | --- | --- | --- | --- | --- | --- | --- |
|  | **1** | **2** | **3** | **4** | **5** |  | **1** | **2** | **3** | **4** | **5** |  | **1** | **2** | **3** | **4** | **5** |
| *Ajania khartensis* | A | A | A | P | P |  | A | A | A | A | A |  | A | A | A | A | A |
| *Ajania tenuifolia* | P | P | P | P | P |  | A | A | A | A | A |  | A | A | A | A | A |
| *Anaphalis lactea* | P | A | P | A | A |  | A | A | A | A | A |  | A | A | A | A | A |
| *Anemone cathayensis* | P | P | P | P | P |  | A | A | A | A | A |  | A | A | A | A | A |
| *Aster tataricus* | P | P | P | P | P |  | P | P | A | P | P |  | A | A | A | A | A |
| *Astragalus membranaceus* | P | P | A | A | A |  | A | A | A | A | A |  | A | A | A | A | A |
| *Bryophytes spp.1* | P | P | P | P | P |  | A | A | A | A | A |  | A | A | A | A | A |
| *Bryophytes spp.2* | A | A | A | A | A |  | P | A | A | A | P |  | A | A | A | A | A |
| *Carex spp.* | P | A | A | A | A |  | P | A | P | P | P |  | P | A | P | P | P |
| *Crepis flexuosa* | A | A | A | A | A |  | A | A | A | A | P |  | A | P | A | A | A |
| *Elymus nutans* | P | P | P | P | P |  | P | A | A | A | P |  | A | A | A | A | A |
| *Gentiana aristata* | A | A | A | A | A |  | P | A | A | A | A |  | A | A | A | A | A |
| *Gentiana spathulifolia* | P | A | P | A | A |  | A | A | A | A | A |  | A | A | A | A | A |
| *Gentiana straminea* | A | A | P | A | P |  | A | A | A | A | A |  | A | A | A | A | A |
| *Gentianella azurea* | P | P | A | A | A |  | A | P | P | A | P |  | A | A | A | A | A |
| *Gentianopsis paludosa* | A | A | A | P | A |  | A | A | A | A | A |  | A | A | A | A | A |
| *Glaux maritima* | P | P | P | P | P |  | A | A | A | A | A |  | A | A | A | A | A |
| *Halerpestes tricuspis* | A | P | A | A | A |  | A | A | A | A | A |  | A | A | A | A | A |
| *Heleocharis dulcis* | A | A | A | A | A |  | A | A | A | A | A |  | P | P | A | A | P |
| *Helictotrichon tibeticum* | A | P | A | A | P |  | A | A | A | A | A |  | A | A | A | A | A |
| *Humulus japonicus* | A | P | A | A | A |  | A | A | A | A | A |  | A | A | A | A | A |
| *Kobresia humilis* | P | P | P | A | P |  | A | A | A | A | A |  | A | A | A | A | A |
| *Lancea tibetica* | P | P | P | P | P |  | P | A | P | A | P |  | A | A | A | A | A |
| *Leontopodium nanum* | A | A | P | P | A |  | A | A | A | A | A |  | A | A | A | A | A |
| *Ligularia sagitta* | A | A | A | A | A |  | P | A | A | A | A |  | A | A | A | A | A |
| *Ligularia virgaurea* | A | A | A | A | A |  | A | P | A | A | A |  | A | A | A | A | A |
| *Medicago ruthenica* | P | P | P | P | P |  | A | A | A | A | A |  | A | A | A | A | A |
| *Morina kokonorica* | A | A | A | P | A |  | A | A | A | A | A |  | A | A | A | A | A |
| *Oxytropis spp.* | A | P | P | A | A |  | A | A | A | A | A |  | A | A | A | A | A |
| *Pedicularis longiflora* | A | A | A | A | A |  | P | A | A | A | P |  | A | A | A | A | A |
| *Pedicularis spp.* | A | P | P | P | P |  | A | A | A | A | A |  | A | A | A | A | A |
| *Poa annua* | A | P | A | P | P |  | A | A | A | A | A |  | A | A | A | P | A |
| *Poaceae spp.* | P | P | P | P | P |  | P | P | P | P | P |  | P | P | P | P | P |
| *Polygonum sibiricum* | A | A | P | P | P |  | A | A | A | A | A |  | A | A | A | A | A |
| *Polygonum viviparum* | A | A | A | A | A |  | A | P | P | P | A |  | A | A | A | A | A |
| *Potentilla anserina* | P | P | P | P | P |  | P | A | A | A | A |  | A | A | A | A | A |
| *Potentilla bifurca* | P | P | A | P | P |  | A | A | A | A | A |  | A | A | A | A | A |
| *Potentilla multifida* | A | P | A | A | A |  | A | A | A | A | A |  | A | A | A | A | A |
| *Potentilla saundersiana* | A | P | P | A | A |  | A | A | A | A | A |  | A | A | A | A | A |
| *Ptilagrostis concinna* | P | P | P | P | P |  | A | P | A | A | A |  | A | A | A | A | A |
| *Ranunculus brotherusii* | P | P | P | P | P |  | A | A | A | A | A |  | A | A | A | A | A |
| *Ranunculus spp.* | A | A | P | A | A |  | P | P | P | P | P |  | A | A | A | A | A |
| *Saussurea romuleifolia* | A | A | A | A | A |  | P | P | P | A | P |  | A | A | A | A | A |
| *Saussurea superba* | P | P | P | P | A |  | A | A | A | A | A |  | A | A | A | A | A |
| *Scirpus distigmaticus* | A | A | A | A | A |  | P | P | P | P | P |  | A | A | A | A | A |
| *Stellaria media* | P | P | P | P | A |  | P | P | P | P | A |  | A | A | A | A | A |
| *Stipa capillata* | A | A | A | A | A |  | P | P | P | A | P |  | A | A | A | A | A |
| *Taraxacum mongolicum* | P | P | P | P | P |  | A | A | A | A | A |  | A | A | A | A | A |
| *Taraxacum spp.* | A | P | A | A | A |  | A | A | A | A | A |  | A | A | A | A | A |
| *Thalictrum alpinum* | P | P | A | A | A |  | P | A | P | A | A |  | A | A | A | A | A |
| *Thalictrum rutifolium* | A | P | P | P | P |  | A | A | A | A | A |  | A | A | A | A | A |
| *Tibetia himalaica* | P | P | P | P | P |  | A | A | A | A | A |  | A | A | A | A | A |
| *Veronica didyma* | A | P | A | P | A |  | A | A | A | A | A |  | A | A | A | A | A |
| *unknown1* | A | A | A | P | A |  | A | A | P | P | P |  | A | A | A | A | P |
| *unknown2* | A | A | A | A | A |  | P | A | P | P | P |  | A | A | A | A | A |

Table S2 Average relative abundances of phyla of bacteria classified with RDP classifier (values represent percentage of total sequences). Overall values were the average of all samples. The “0.00” value means less than 0.01.

| Phylum | Overall | Alpine meadow | Marsh meadow | Marsh |
| --- | --- | --- | --- | --- |
| *Actinobacteria* | 21.09 | 33.21 | 18.01 | 12.05 |
| *Alphaproteobacteria* | 16.87 | 17.80 | 24.51 | 8.30 |
| *Betaproteobacteria* | 13.10 | 7.82 | 12.88 | 18.59 |
| *Deltaproteobacteria* | 9.40 | 4.39 | 10.16 | 13.66 |
| *Acidobacteria* | 8.25 | 10.41 | 9.87 | 4.46 |
| *Bacteroidetes* | 7.55 | 4.67 | 5.07 | 12.91 |
| *Chloroflexi* | 7.40 | 5.98 | 6.71 | 9.53 |
| *Gammaproteobacteria* | 4.88 | 4.19 | 3.46 | 6.98 |
| *Firmicutes* | 2.75 | 2.40 | 1.77 | 4.07 |
| *Planctomycetes* | 2.19 | 2.89 | 2.07 | 1.59 |
| *Gemmatimonadetes* | 1.91 | 3.83 | 1.41 | 0.50 |
| *Chlorobi* | 1.10 | 0.11 | 0.74 | 2.44 |
| *Nitrospirae* | 0.78 | 0.75 | 0.82 | 0.76 |
| *WS3* | 0.44 | 0.43 | 0.37 | 0.52 |
| *Verrucomicrobia* | 0.30 | 0.10 | 0.30 | 0.51 |
| *NC10* | 0.24 | 0.00 | 0.37 | 0.35 |
| *Armatimonadetes* | 0.23 | 0.40 | 0.11 | 0.19 |
| *Cyanobacteria* | 0.21 | 0.07 | 0.17 | 0.41 |
| *Elusimicrobia* | 0.20 | 0.11 | 0.36 | 0.13 |
| *OP8* | 0.20 | 0.00 | 0.03 | 0.55 |
| *Spirochaetes* | 0.14 | 0.00 | 0.06 | 0.35 |
| *OP3* | 0.11 | 0.01 | 0.16 | 0.16 |
| *Proteobacteria;other* | 0.11 | 0.09 | 0.10 | 0.14 |
| *Fibrobacteres* | 0.06 | 0.02 | 0.03 | 0.13 |
| *TM7* | 0.06 | 0.04 | 0.03 | 0.10 |
| *TM6* | 0.05 | 0.04 | 0.08 | 0.03 |
| *NKB19* | 0.04 | 0.03 | 0.02 | 0.07 |
| *GOUTA4* | 0.04 | 0.00 | 0.03 | 0.08 |
| *Fusobacteria* | 0.02 | 0.00 | 0.00 | 0.05 |

Table S3 Average relative abundances of phyla of archaea classified with RDP classifier (values represent percentage of total sequences). Overall values were the average of all samples.

| Phylum | Overall | Alpine meadow | Marsh meadow | Marsh |
| --- | --- | --- | --- | --- |
| *Thaumarchaeota* | 49.34 | 92.45 | 43.82 | 11.76 |
| *Euryarchaeota* | 30.62 | 5.33 | 21.96 | 64.58 |
| *Methanomicrobia* | 22.31 | 4.34 | 10.76 | 51.82 |
| *Methanobacteria* | 6.33 | 0.71 | 9.38 | 8.89 |
| *Thermoplasmata* | 1.99 | 0.28 | 1.80 | 3.88 |
| *Crenarchaeota* | 17.43 | 1.83 | 29.32 | 21.14 |
| *Parvarchaeota* | 2.60 | 0.38 | 4.91 | 2.52 |

Table S4 Average relative abundances of taxa of eukaryotes classified with RDP classifier (values represent percentage of total sequences). Overall values were the average of all samples. The “0.00” value means less than 0.01.

| Phylum | Overall | Alpine meadow | Marsh meadow | Marsh |
| --- | --- | --- | --- | --- |
| *Fungi* | 25.22 | 46.35 | 14.93 | 14.39 |
| *Ascomycota* | 12.73 | 22.99 | 7.64 | 7.55 |
| *Basidiomycota* | 8.07 | 17.45 | 4.70 | 2.04 |
| *Chytridiomycota* | 1.70 | 1.57 | 1.29 | 2.25 |
| *Glomeromycota* | 0.96 | 2.51 | 0.36 | 0.02 |
| *Blastocladiomycota* | 0.02 | 0.03 | 0.02 | 0.00 |
| *Other* | 1.75 | 1.80 | 0.91 | 2.54 |
| *Alveolata* | 22.04 | 13.55 | 24.99 | 27.58 |
| *Metazoa* | 16.34 | 9.32 | 17.19 | 22.51 |
| *environmental* | 10.57 | 7.24 | 11.71 | 12.76 |
| *Rhizaria* | 9.85 | 8.38 | 12.67 | 8.52 |
| *Other* | 9.72 | 10.66 | 11.67 | 6.82 |
| *stramenopiles* | 3.80 | 2.09 | 3.86 | 5.46 |
| *Amoebozoa* | 1.88 | 2.09 | 2.26 | 1.30 |
| *Ichthyosporea* | 0.17 | 0.21 | 0.18 | 0.13 |
| *Euglenozoa* | 0.17 | 0.04 | 0.14 | 0.32 |
| *Apusozoa* | 0.08 | 0.05 | 0.13 | 0.05 |
| *Choanoflagellida* | 0.07 | 0.00 | 0.15 | 0.05 |
| *Jakobida* | 0.03 | 0.00 | 0.03 | 0.05 |

Table S5 Pairwise comparison of microbial community examined by ADONIS (P<0.05, permutation=999).

| tested pair | bacteria | |  | archaea | |  | eukaryotes | |
| --- | --- | --- | --- | --- | --- | --- | --- | --- |
|  | F | P |  | F | P |  | F | P |
| Alpine meadow-Marsh meadow | 3.99 | 0.012 |  | 12.9 | 0.008 |  | 4.61 | 0.007 |
| Marsh meadow-Marsh | 7.41 | 0.013 |  | 14.2 | 0.01 |  | 5.51 | 0.007 |
| Alpine meadow-Marsh | 7.41 | 0.008 |  | 14.2 | 0.006 |  | 5.51 | 0.012 |

Table S6: Percentage (R2) of microbial community variation explained by different habitats examined by ADONIS (P<0.05, Permutation = 999).

| Microbe | R^2^ | P |
| --- | --- | --- |
| Bacteria | 0.466 | 0.001 |
| Archaea | 0.753 | 0.001 |
| Eukaryotes | 0.433 | 0.001 |

Table S7 The environmental factors that significantly correlated with microbial communities determined by Mantel Tests between (P<0.05, permutation=999). The top four correlation coefficients are listed in bold. For abbreviations, see Table 1.

| variable | bacteria | |  | archaea | |  | eukaryotes | |
| --- | --- | --- | --- | --- | --- | --- | --- | --- |
|  | r | P |  | r | P |  | r | P |
| pH | **0.228** | 0.02 |  | **0.197** | 0.022 |  | **0.412** | 0.002 |
| SM | **0.792** | 0.001 |  | **0.620** | 0.002 |  | **0.834** | 0.001 |
| TC | **0.582** | 0.001 |  | **0.684** | 0.001 |  | **0.683** | 0.001 |
| TN | **0.525** | 0.003 |  | **0.639** | 0.001 |  | **0.575** | 0.001 |
| C/N | **0.714** | 0.001 |  | **0.668** | 0.001 |  | **0.727** | 0.001 |
| DOC | **0.400** | 0.001 |  | **0.436** | 0.001 |  | **0.525** | 0.001 |
| DON | **0.388** | 0.008 |  | **0.374** | 0.003 |  | **0.468** | 0.004 |
| NH_4_^+^ | **0.225** | 0.036 |  | 0.140 | 0.114 |  | **0.187** | 0.047 |
| NO_3_^-^ | 0.027 | 0.293 |  | 0.031 | 0.324 |  | -0.094 | 0.812 |

Table S8 Variance of microbial community explained by soil characteristics (A: bacteria; B: archaea; C: eukaryotes) calculated by DistLM forward3 (P<0.05, permutation=999). Variables with significant values were listed in bold. SS: sum of square; prop: proportion of explained variance of corresponding soil variable; cumul: cumulative proportion of explained variance. For soil variable abbreviations, see Table 1.

| A | Variable | SS | F | P | prop | cumul |
| --- | --- | --- | --- | --- | --- | --- |
|  | **SM** | 12768 | 5.16 | **0.001** | 0.28 | 0.28 |
|  | **TN** | 7424 | 3.60 | **0.001** | 0.17 | 0.45 |
|  | C/N | 2570 | 1.28 | 0.073 | 0.06 | 0.51 |
|  | DON | 2240 | 1.13 | 0.321 | 0.05 | 0.56 |
|  | NO_3_^-^ | 2126 | 1.08 | 0.418 | 0.05 | 0.60 |
|  | DOC | 2148 | 1.10 | 0.393 | 0.05 | 0.65 |
|  | TC | 2141 | 1.11 | 0.410 | 0.05 | 0.70 |
|  | pH | 1958 | 1.02 | 0.461 | 0.04 | 0.74 |
|  | NH_4_^+^ | 1818 | 0.94 | 0.534 | 0.04 | 0.78 |

| B | Variable | SS | F | P | prop | cumul |
| --- | --- | --- | --- | --- | --- | --- |
|  | **C/N** | 16580 | 9.65 | **0.001** | 0.43 | 0.43 |
|  | **TC** | 9163 | 8.34 | **0.001** | 0.24 | 0.66 |
|  | **DOC** | 3150 | 3.46 | **0.001** | 0.08 | 0.74 |
|  | **TN** | 1661 | 1.98 | **0.015** | 0.04 | 0.79 |
|  | SM | 1248 | 1.58 | 0.088 | 0.03 | 0.82 |
|  | pH | 949 | 1.23 | 0.253 | 0.02 | 0.84 |
|  | DON | 1044 | 1.42 | 0.190 | 0.03 | 0.87 |
|  | NH_4_^+^ | 756 | 1.04 | 0.434 | 0.02 | 0.89 |
|  | NO_3_^-^ | 431 | 0.55 | 0.792 | 0.01 | 0.90 |

| C | Variable | SS | F | P | prop | cumul |
| --- | --- | --- | --- | --- | --- | --- |
|  | **SM** | 9344 | 4.69 | **0.001** | 0.28 | 0.28 |
|  | **TC** | 4459 | 2.52 | **0.001** | 0.13 | 0.41 |
|  | DON | 2326 | 1.36 | 0.059 | 0.07 | 0.48 |
|  | TN | 1944 | 1.15 | 0.270 | 0.06 | 0.54 |
|  | pH | 1764 | 1.05 | 0.448 | 0.05 | 0.60 |
|  | NH_4_^+^ | 1757 | 1.05 | 0.471 | 0.05 | 0.65 |
|  | DOC | 1691 | 1.02 | 0.468 | 0.05 | 0.70 |
|  | C/N | 1719 | 1.04 | 0.466 | 0.05 | 0.75 |
|  | NO_3_^-^ | 1266 | 0.72 | 0.667 | 0.04 | 0.79 |

Table S9 Changes in microbial diversity across vegetation types. Phylogenetic diversity (Faith’s PD) and observed species (OTUs) of bacteria, archaea and eukaryote were calculated using random selections of 2800, 1110 and 640 sequences per sample, respectively. Values are listed in the form of “mean (standard error)”. Different letters represent significant differences.

| Diversity Index | Habitat | bacteria | archaea | eukaryotes |
| --- | --- | --- | --- | --- |
| PD | Alpine meadow | 117 (1.6)^b^ | 7.21 (2.25)^b^ | 2.48 (0.10)^b^ |
|  | Marsh meadow | 129 (1.7)^a^ | 16.8 (1.28)^a^ | 2.71 (0.16)^b^ |
|  | Marsh | 123 (3.5)^ab^ | 20.2 (1.19)^a^ | 3.42 (0.11)^a^ |
| OTUs | Alpine meadow | 1708 (24)^a^ | 245 (32)^b^ | 240 (14)^a^ |
|  | Marsh meadow | 1740 (11)^a^ | 319 (20)^ab^ | 272 (7.9)^a^ |
|  | Marsh | 1606 (43)^b^ | 382 (18)^a^ | 268 (8.4)^a^ |

Table S10 Pearson correlations (r) between microbial diversity (Faith’s PD and OTUs) and soil characteristics. Significant values are listed in bold. For abbreviations, see Table 1.

| r (P<0.05) | PD | | |  | OTUs | | |
| --- | --- | --- | --- | --- | --- | --- | --- |
|  | bacteria | archaea | eukaryotes |  | bacteria | archaea | eukaryotes |
| pH | -0.486 | -0.498 | -0.324 |  | 0.074 | -0.394 | -0.349 |
| SM | 0.28 | **0.791** | **0.753** |  | -0.503 | **0.665** | 0.455 |
| TC | **0.583** | **0.665** | 0.326 |  | -0.008 | 0.451 | **0.544** |
| TN | **0.556** | **0.534** | 0.159 |  | 0.084 | 0.308 | 0.512 |
| C/N | 0.441 | **0.855** | **0.763** |  | -0.324 | **0.772** | 0.469 |
| DOC | 0.201 | **0.788** | **0.735** |  | -0.443 | **0.773** | **0.636** |
| DON | 0.139 | **0.564** | 0.476 |  | -0.448 | 0.407 | 0.318 |
| NO_3_^-^ | -0.071 | -0.221 | -0.312 |  | 0.117 | -0.313 | 0.285 |
| NH_4_^+^ | 0.27 | 0.4 | 0.212 |  | -0.148 | 0.259 | 0.009 |

Table S11 Gene number of representative KEGG pathways in different habitats determined by indicator species analysis. “Site” denotes which habitat the pathway belongs to.

| **Pathway category** | **Site** | **Alpine meadow** | **Marsh meadow** | **Marsh** |
| --- | --- | --- | --- | --- |
| Cellular Processes | Alpine meadow | 37.8 | 4.2 | 3 |
| Environmental Information Processing | Alpine meadow | 38.2 | 4.4 | 3 |
| Genetic Information Processing | Alpine meadow | 508.8 | 178.6 | 51 |
| Metabolism | Alpine meadow | 151.8 | 68.2 | 13.4 |
| Organismal Systems | Alpine meadow | 9.6 | 1.4 | 0 |
| Cellular Processes | Marsh meadow | 24 | 35.2 | 11 |
| Metabolism | Marsh meadow | 441.4 | 658.8 | 175.4 |
| Organismal Systems | Marsh meadow | 909 | 1306.4 | 346.8 |
| Metabolism | Marsh | 0 | 0.4 | 2 |
| Organismal Systems | Marsh | 1 | 13 | 25.6 |

Table S12 Overall proportion of beta NTI value that belongs to different ecological processes

Table S13 The average value of between habitats beta NTI. Different letters in bracket indicate significant difference tested by ANOVA. AM: alpine meadow, MM: marsh meadow, MA: marsh

| Proportion (%) | bacteria | archaea | eukaryotes |
| --- | --- | --- | --- |
| < -2 | 51.43 | 51.28 | 20.88 |
| > +2 | 33.33 | 12.82 | 0.00 |
| -2 < & < +2 | 15.24 | 35.90 | 79.12 |


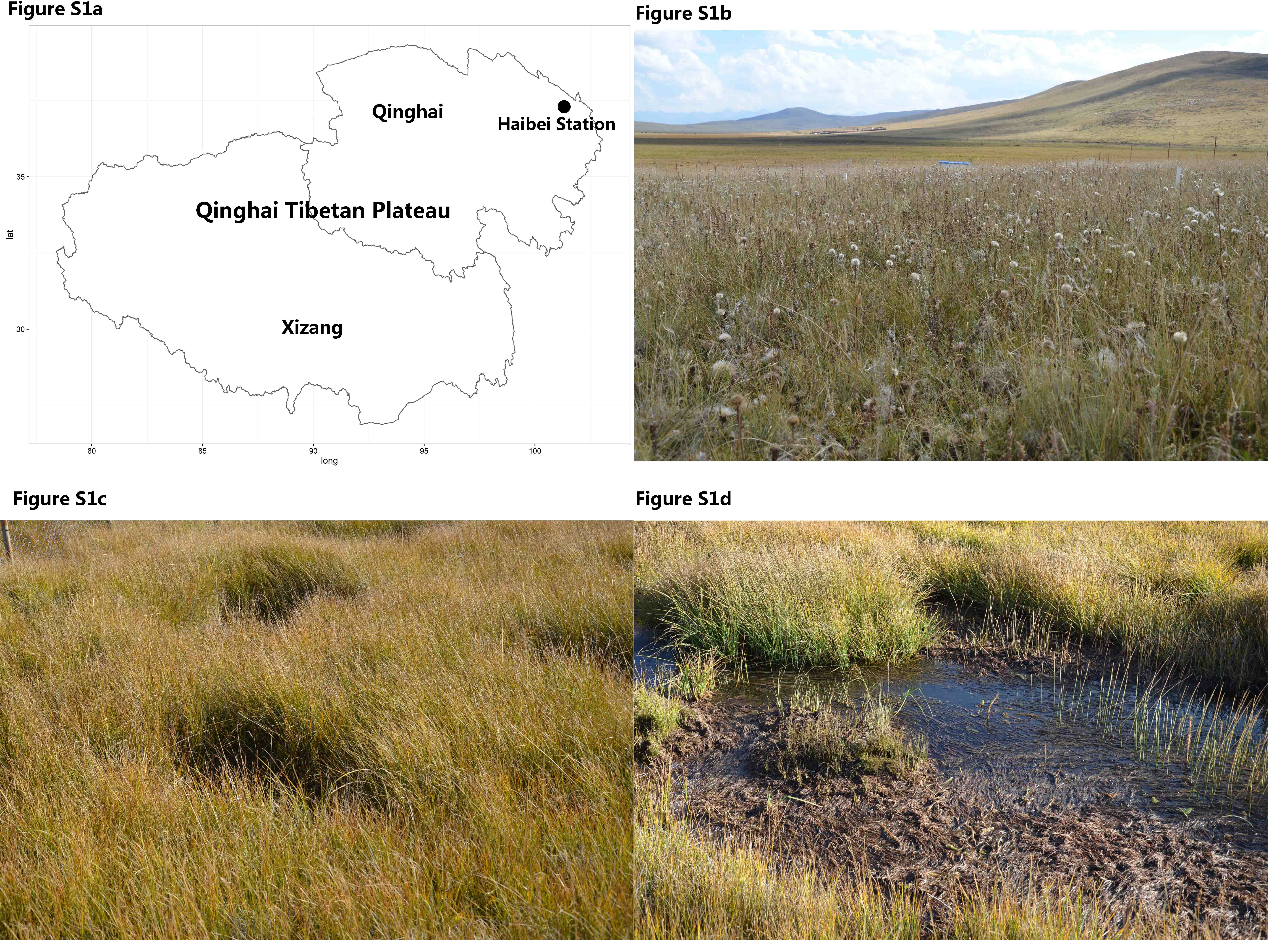
Figure S1: Map showing the position of Haibei Station (a) and photos showing the three habitats with different soil water availability. The map was generated using package maptools and ggplot2 in R 3.1.0 (https://www.r-project.org/). b: Alpine meadow, c: Marsh meadow, d: Marsh

| Pair | bacteria | archaea | eukaryotes |
| --- | --- | --- | --- |
| AM-MM | -0.76 (a) | -7.65 (b) | 0.37 (a) |
| AM-MA | -15.08 (c) | -21.48 (c) | -5.21 (b) |
| MM-MA | -4.06 (b) | -1.59 (a) | -1.21 (a) |


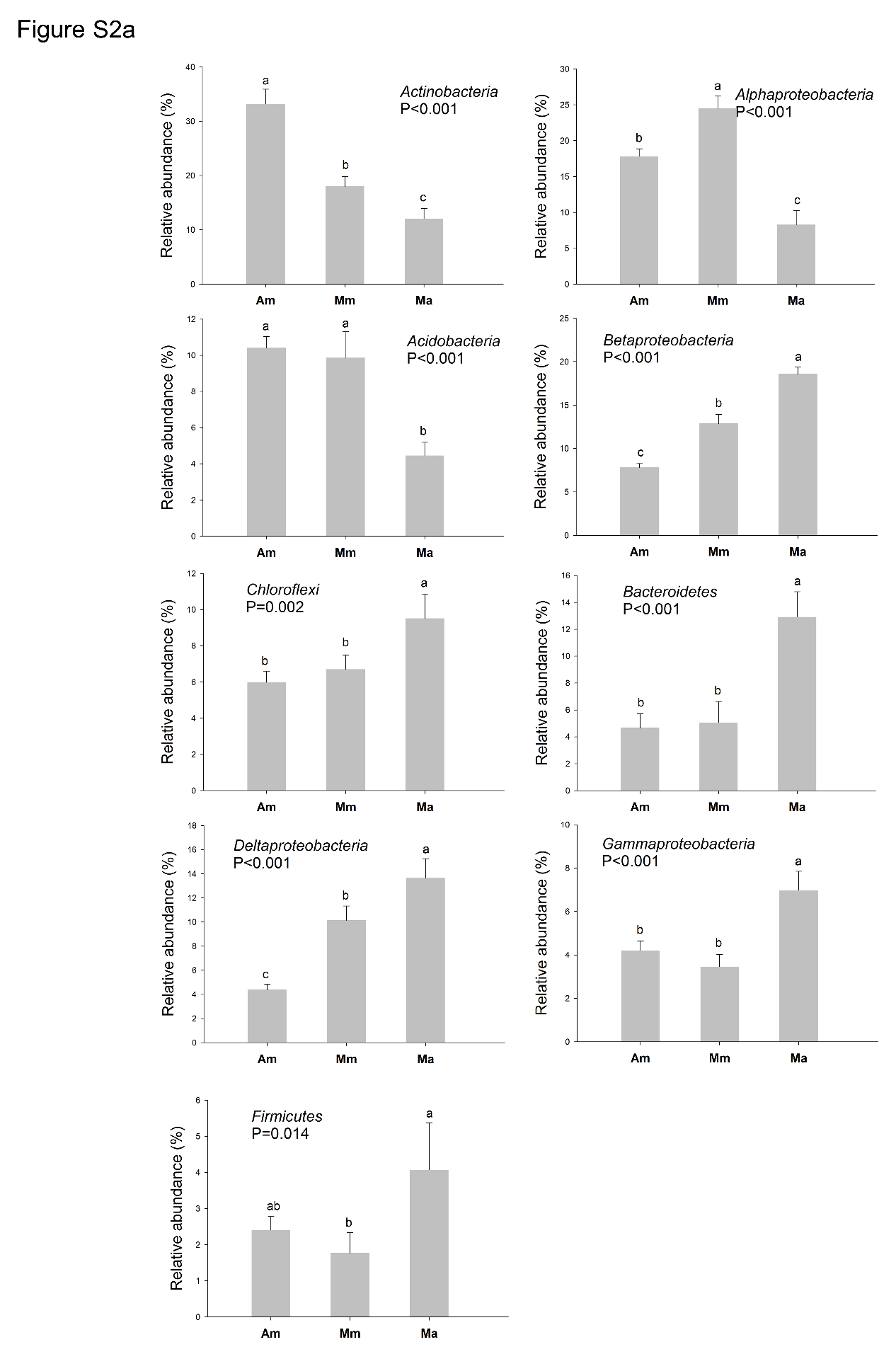
Figure S2: Multiple comparison of dominant phyla in bacteria (a), archaea (b) and eukaryotes (c). Am: Alpine meadow; Mm: Marsh meadow; Ma: Marsh


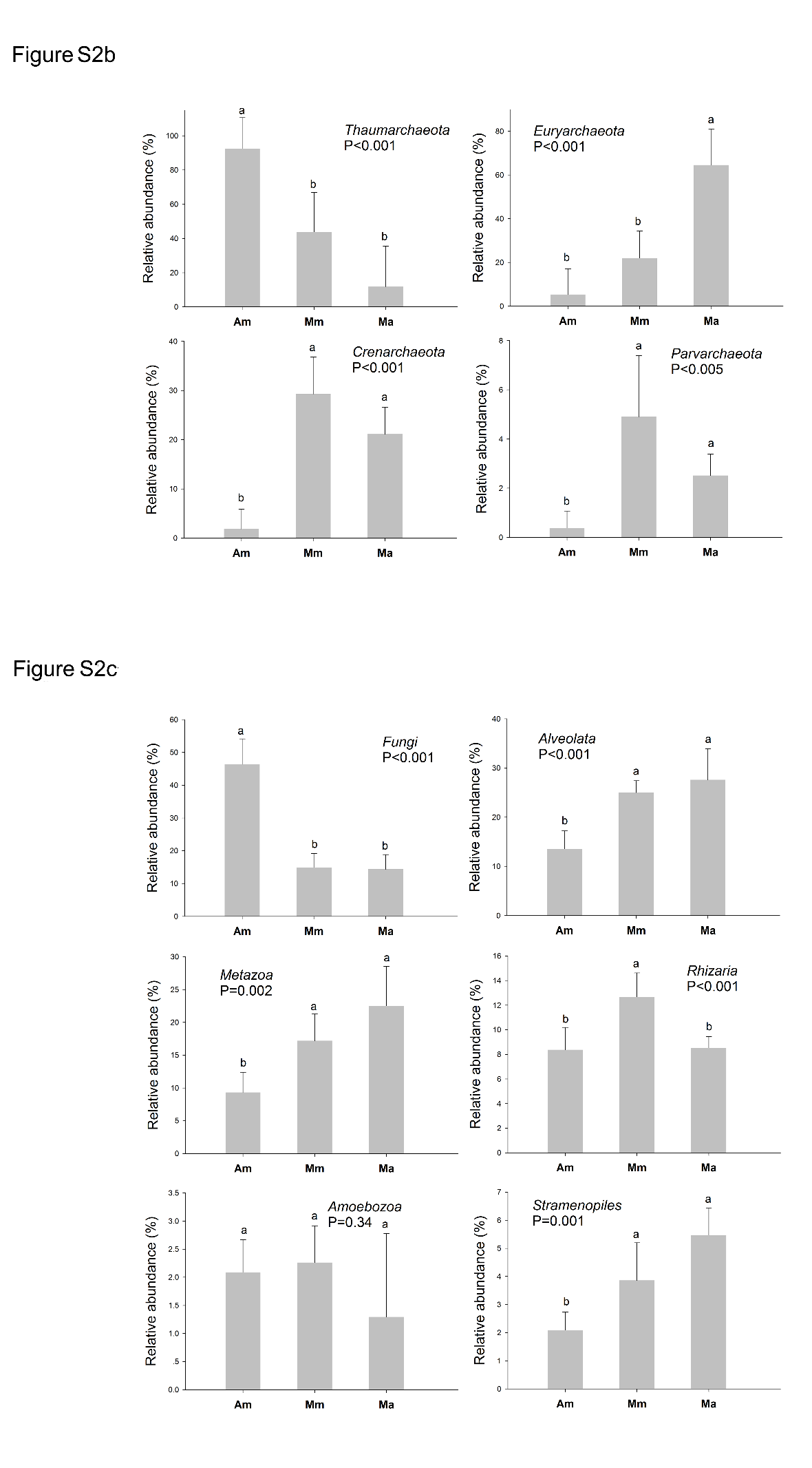


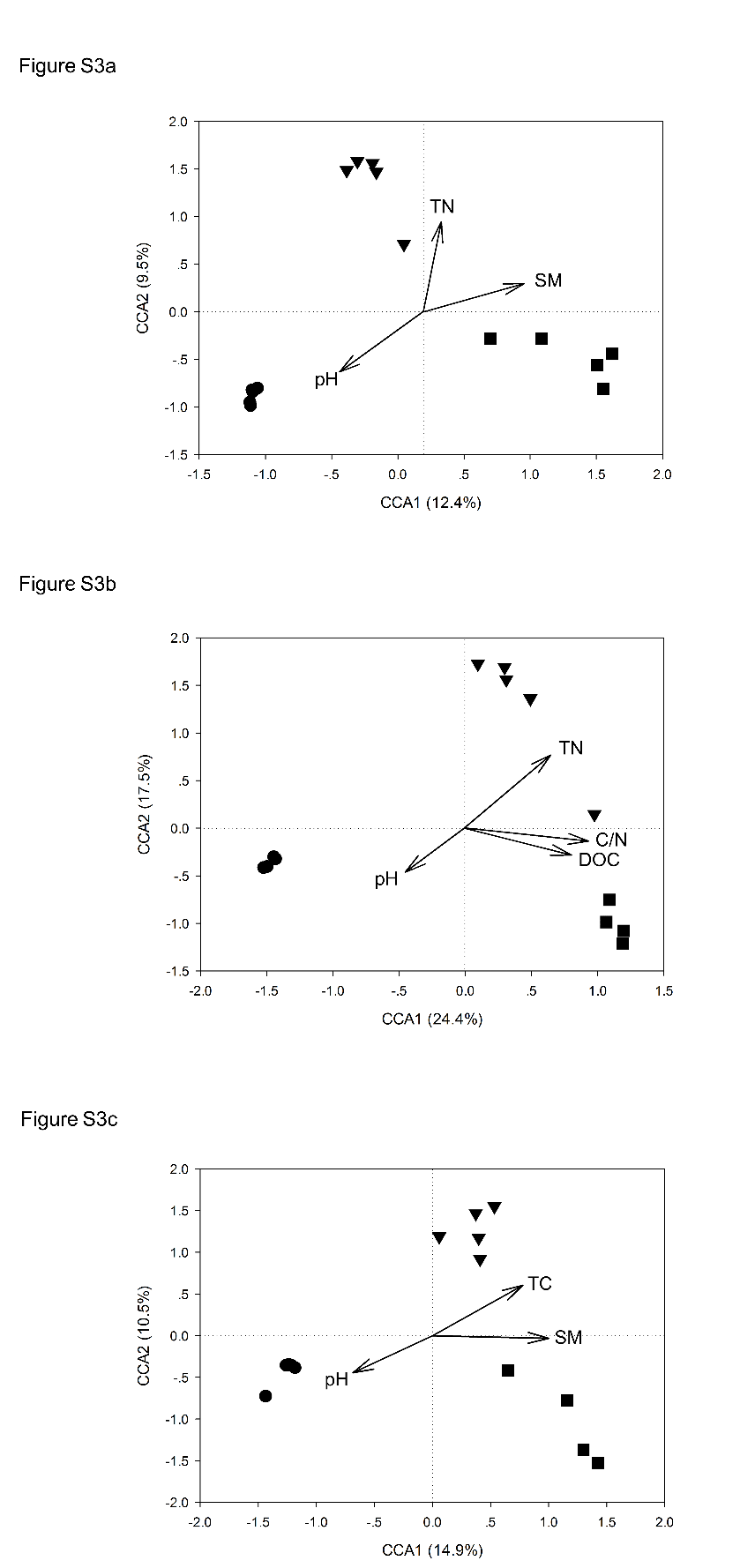
Figure S3: Canonical correspondence analysis (CCA) of bacterial (A), archaeal (B) and eukaryotic (C) communities (●Alpine meadow; ▲Marsh meadow; ■Marsh). For abbreviations, see Table 1


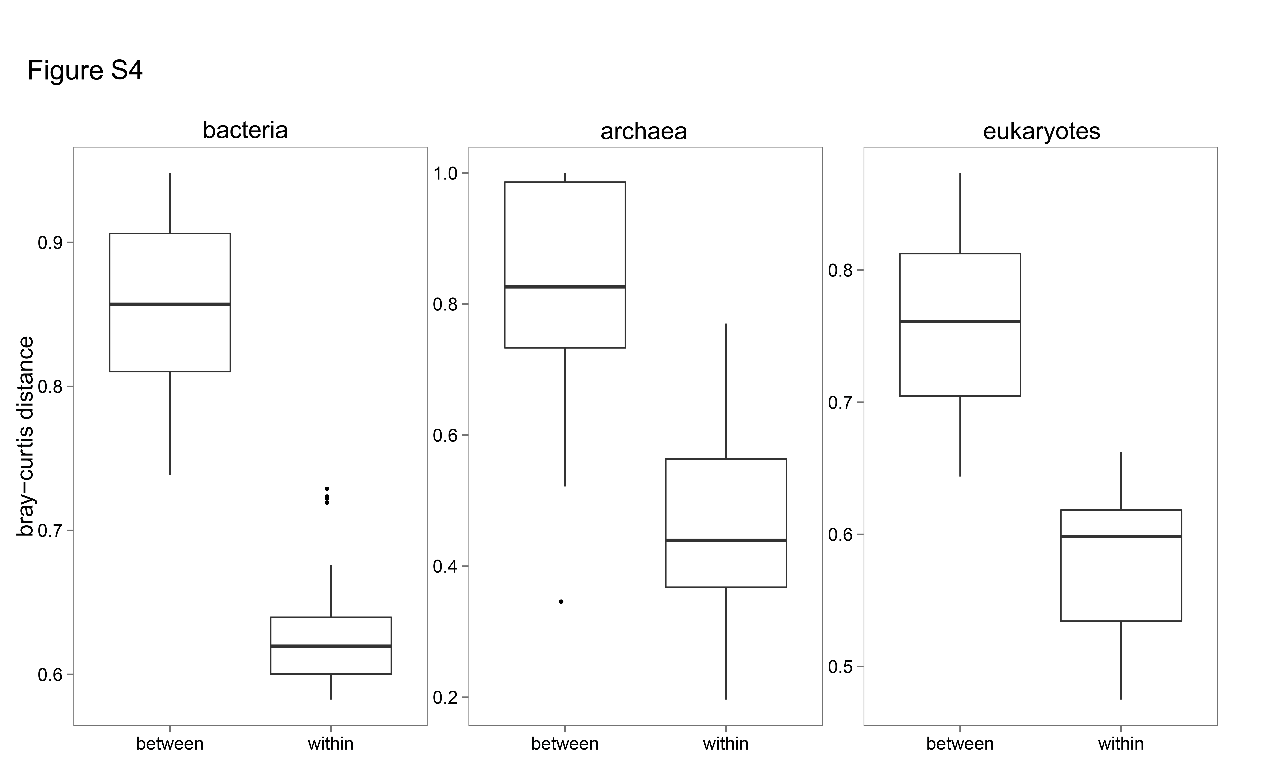
Figure S4: Grouped microbial bray-curtis dissimilarity distribution. Significant difference was found by t-test (P<0.001) between two groups in all microbes. Inner lines represent the group median; edges of the box represent the upper and lower quartile. Whiskers extend to the group highest value or 1.5×IQR (Interquartile Range). Points beyond whiskers are outliers. Between: plot pairs that belong to different habitats; within: plot pairs in the same habitat.


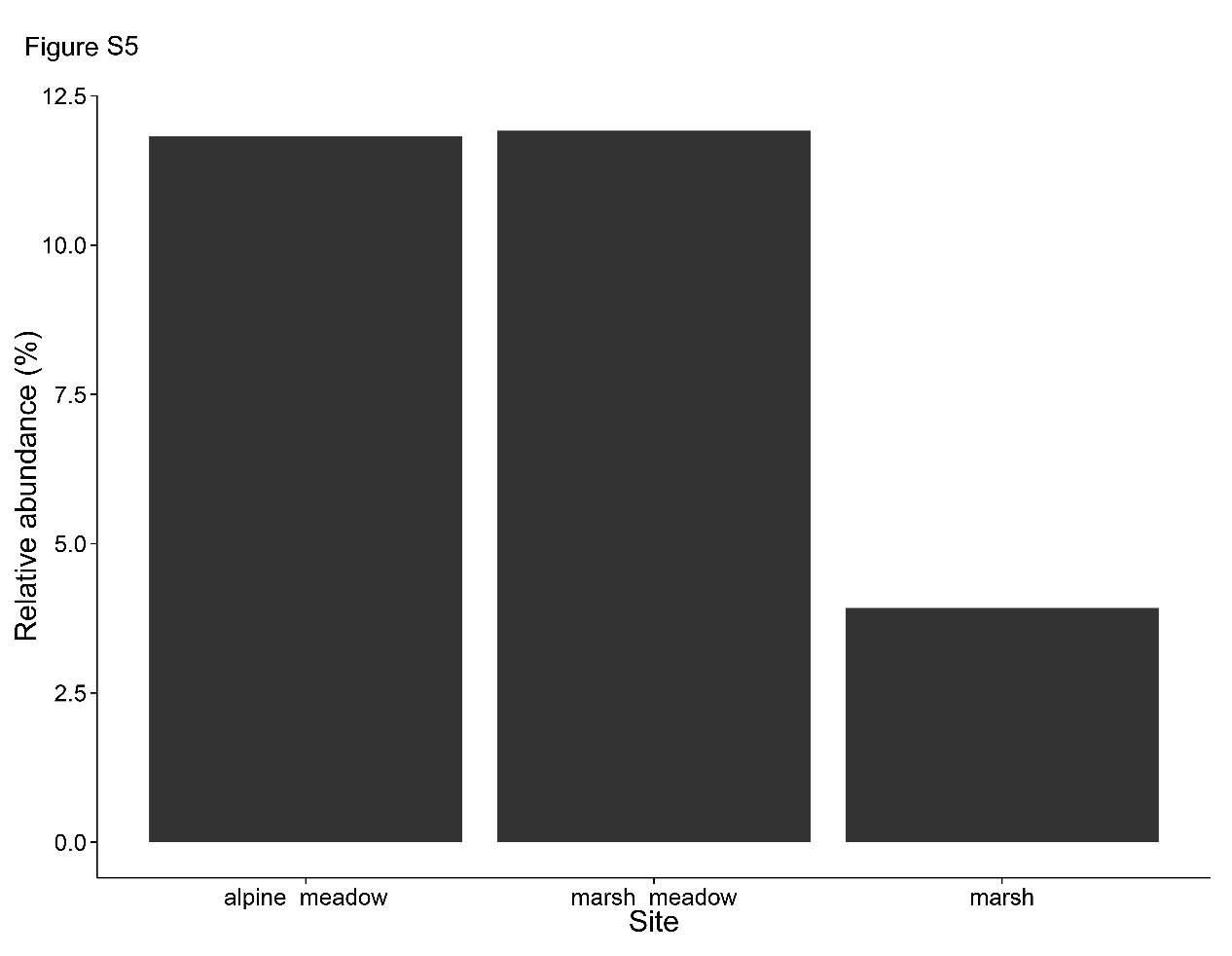
Figure S5: Relative abundance of family Rhizobiales in bacteria among different habitats


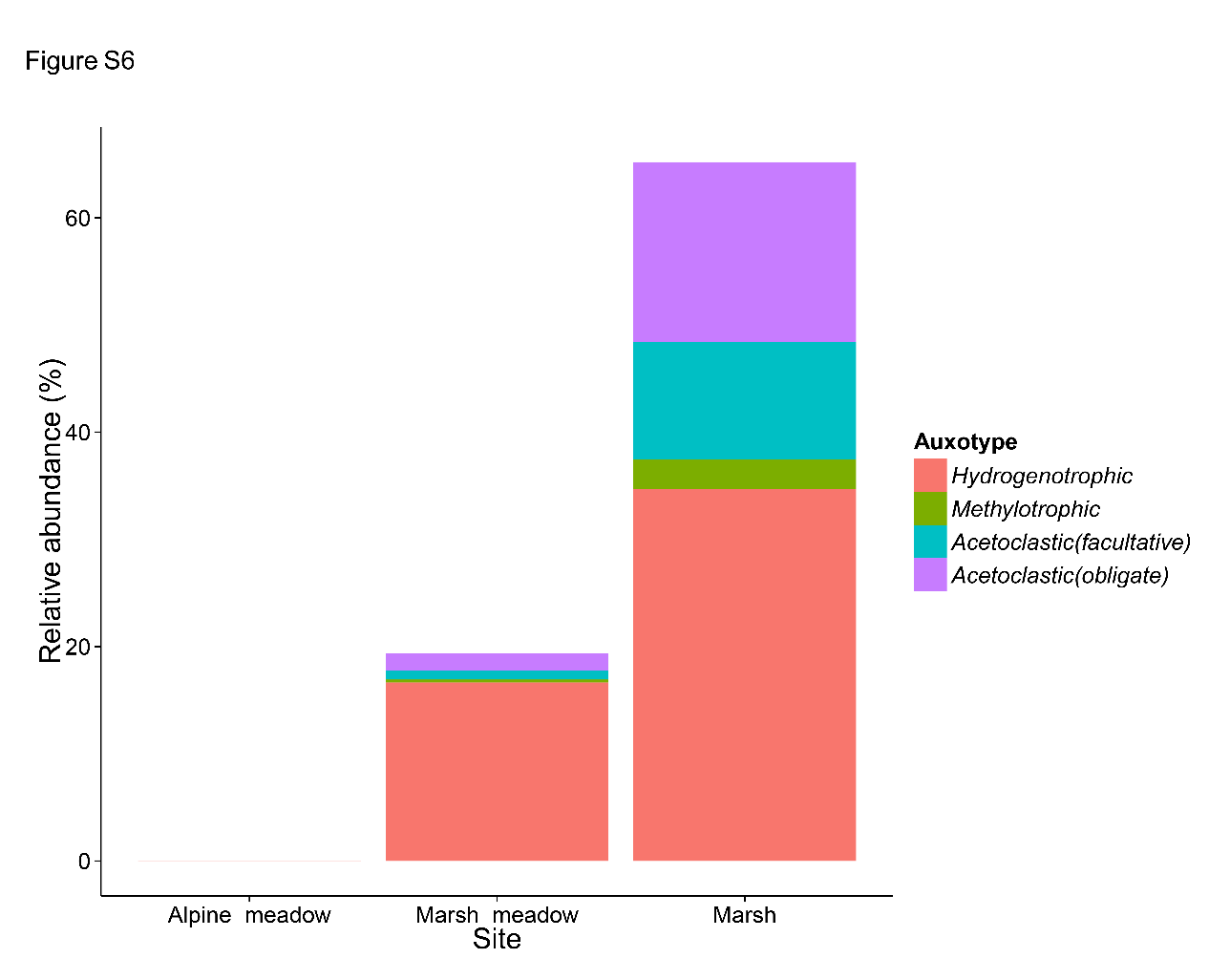
Figure S6: Relative abundance of functional methanogenic archaeal groups among different habitats

Figure S7 Scatter plot showing the distribution pattern of beta NTI between habitats. For abbreviations, see Fig. S2


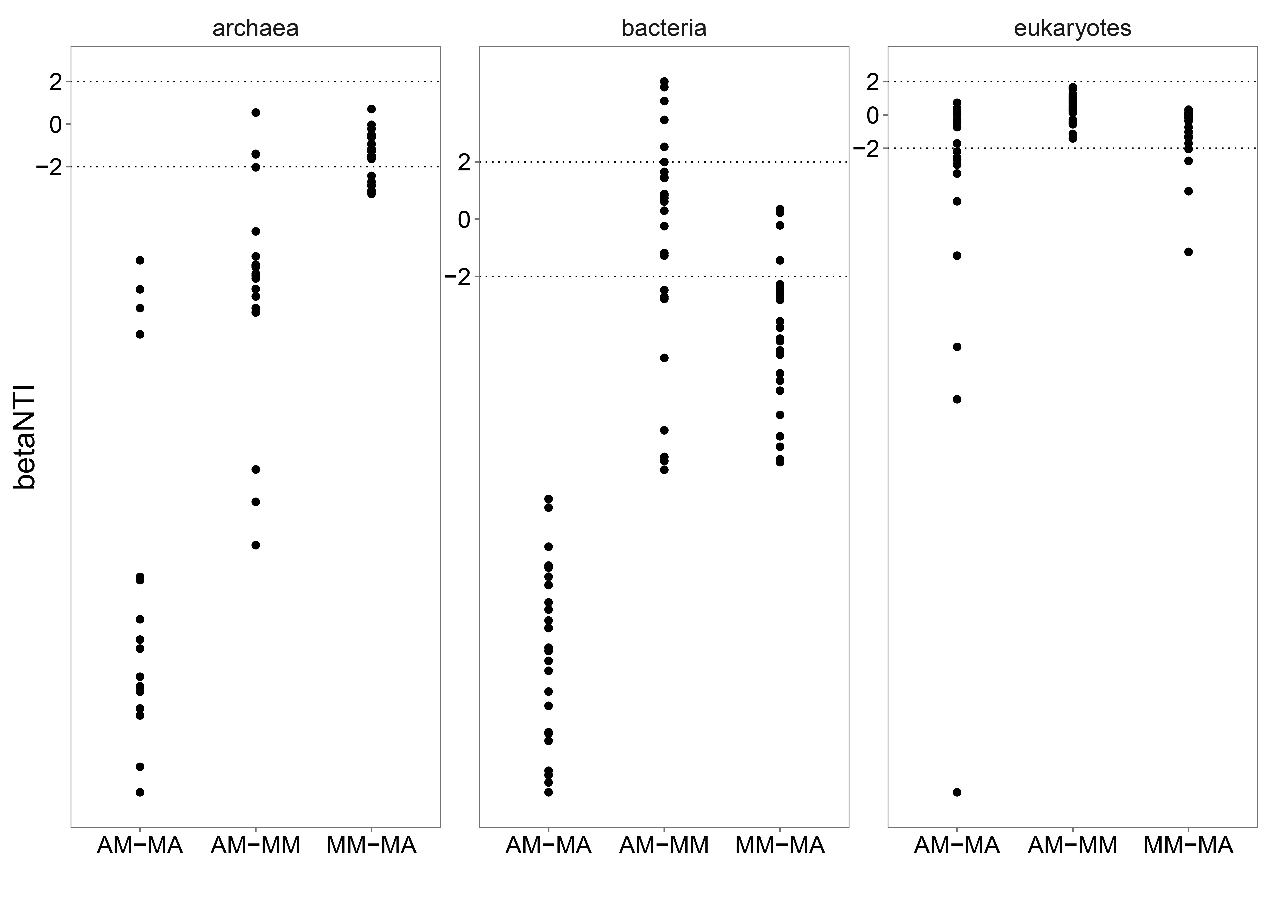

Supplement: Supplementary Information [file srep46407-s1.doc]
